# Trends in *Strongyloides stercoralis* Faecal Larvae Detections in the Northern Territory, Australia: 2002 to 2012

**DOI:** 10.3390/tropicalmed2020018

**Published:** 2017-06-19

**Authors:** Johanna K. Mayer-Coverdale, Amy Crowe, Pamela Smith, Robert W. Baird

**Affiliations:** 1 Department of Microbiology, Pathology Queensland, Royal Brisbane and Women’s Hospital, Queensland 4001, Australia; johannak.mayer@gmail.com; 2 Infectious Diseases Physician, St Vincent’s Hospital, 41 Victoria Pde, Fitzroy 3065, Australia; amy.crowe@svha.org.au; 3 Microbiology, Territory Pathology, Royal Darwin Hospital, Tiwi 0810, Australia; pam.smith@nt.gov.au

**Keywords:** Strongyloides, anaemia, eosinophilia, polyparasitism, indigenous, Northern Territory, Australia

## Abstract

*Strongyloides stercoralis* is a soil-transmitted helminth (STH) endemic to tropical and subtropical areas. We reviewed the temporal detection trends in patients with *S. stercoralis* larvae present in faecal samples, in Northern Territory (NT) Government Health facilities, between 2002 and 2012. This was a retrospective observational study of consecutive patients with microbiologically confirmed detection of *S. stercoralis* in faeces. The presence of anaemia, eosinophilia, polyparasitism, and geographic and demographic data, were included in the assessment. *S. stercoralis* larvae were present in 389 of 22,892 faecal samples (1.7%) collected across the NT over 11 years, examined by microscopy after formol ethyl acetate concentration. 97.7% of detections were in Indigenous patients. Detections, by number, occurred in a biphasic age distribution. Detections per number of faecal samples collected, were highest in the 0–5 year age group. Anaemia was present in 44.8%, and eosinophilia in 49.9% of patients. Eosinophilia was present in 65.5% of the ≤5 age group, compared to 40.8% of >5 year age (*p* < 0.0001). Polyparasitism was present in 31.4% of patients. There was an overall downward trend in larvae detections from 2.64% to 0.99% detections/number of faecal samples year between 2002 and 2012, consistent with the trends observed for other local STHs. *S. stercoralis* remains an important NT-wide pathogen.

## 1. Introduction

*Strongyloides stercoralis* is a soil-transmitted helminth (STH) endemic to tropical and subtropical regions around the world. There are very wide estimates, between 3 and 100 million people [1], of the number of persons infected with *S. stercoralis* globally, because the true incidence is difficult to determine as many infections occur in regions with economic and health hardware disadvantage, and poor access to laboratory diagnostic services.

*S. stercoralis* infection has been estimated to occur disproportionately in Australian Indigenous people living in the northern parts of Australia, not only compared to the rest of Australia, but also to ecologically similar regions around the world [2,3,4,5,6]. Indigenous Australians experience substantially poorer health, social, and economic outcomes compared to other Australians, including overcrowding and poor sanitary conditions [7], both of which are of importance in the transmission of *S. stercoralis*. The Northern Territory (NT) is a large but sparsely populated area covering 1.5 million square kilometers, with numerous remote Indigenous communities. Cases of intestinal obstruction complicating *S. stercoralis* infection in children have been documented in the NT since the 1970s [2], and a 1993 study documented *S. stercoralis* endemicity in Indigenous patients over a 12-month period [3]. An association between HTLV-1 and *S. stercoralis* has been documented in central Australia where HTLV-1 is endemic [4]. *S. stercoralis* detections have been described across the tropical and subtropical areas of Western Australia and Queensland, including to latitudes below the Tropic of Capricorn (latitude 23° S) [5,6]. 

*S. stercoralis* has a complex life cycle [8]. The most common way of becoming infected is by direct contact with soil, as the filariform larvae penetrate the host skin from soil that is contaminated with *S. stercoralis* larvae. High risk activities include walking with bare feet, direct contact with human waste or sewage, as well as occupations such as farming. The larvae migrate via the venous circulation to the lungs, where they ascend the bronchial tree and eventually are swallowed, thus entering the digestive tract. Once established in the small bowel they become adults. The female worms produce eggs by parthenogenesis, which subsequently hatch in the small bowel, as rhabditiform larvae, (non-infectious), then are deposited, and develop to filariform (infectious) larvae in the soil over several days. Larvae that are not passed in the faeces have the ability to develop in the bowel to filariform larvae, that can either penetrate the bowel wall directly, or the perianal mucosa repeating the infective cycle potentially indefinitely. This process of auto-infection is characteristic of *S. stercoralis* and can lead to hyperinfection even in individuals who have not been in endemic areas for decades. Clinically, strongyloidiasis may present as a range of syndromes, ranging from asymptomatic or mild abdominal discomfort, to Crohn’s-like colitis, primarily pulmonary symptoms (Loeffler’s syndrome), cutaneous larva currens, and disseminated infection with high associated mortality [9]. The latter is particularly associated with immunosuppressed patients, especially those taking corticosteroids. The ability to establish ongoing, potentially life-long infection coupled with the risk of dissemination that is associated with high mortality makes screening, and in some instances empiric treatment, for *S. stercoralis* infection in people who are about to undergo immunosuppression essential, not only in endemic areas, but also in selected populations such as migrants from endemic regions and refugees [10]. 

Our aim was to describe the temporal trends in *S. stercoralis* larvae detection in faecal samples collected in NT Government Health facilities over a period of 11 years, and review associated demographic and laboratory features, in this population. 

## 2. Results

Between 2002 and 2012, 22,892 faecal samples were assessed for ova, cysts, and parasites (OCP) across NT Government Health facilities. The number of annual faecal microscopy samples examined remained relatively constant throughout this time period, approximately 2000 samples/year, though 2578 and 2919 faecal microscopy and concentrates were examined in 2011 and 2012, respectively. *S. stercoralis* larvae were detected in 389 (1.7%) of all faecal samples. Eleven patients had more than one episode, with one patient having three documented episodes. 

Demographic and laboratory characteristics are summarised in Table 1.

Overall 381 (97.7%) patients were Indigenous, and 164 (42.2%) were five years old or younger. In the ≤5 year-old group, 163 (99.4%) patients were Indigenous, while in the >5 year-old group 218 (96.9%) were Indigenous. In total, 216 (55.5%) patients were male and 173 (44.5%) female. The male to female ratio in the ≤5 year-old group was 79 (48.2%) male, and 85 (51.8%) female compared to 137 (60.9%) and 88 (39.1%) respectively in the older group. 

**Figure 1:**
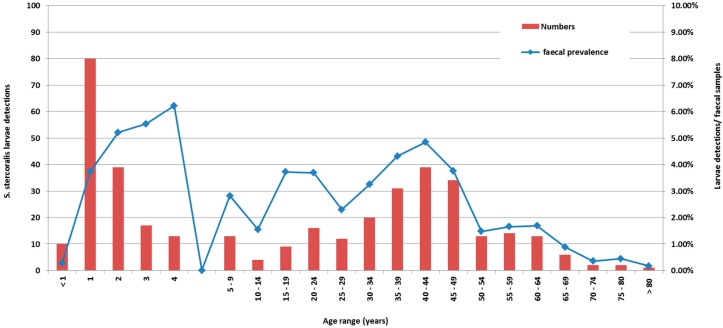
*S. stercoralis* larvae detections by age and diagnostic prevalence in the Northern Territory, 2002–2012.

**Figure 2:**
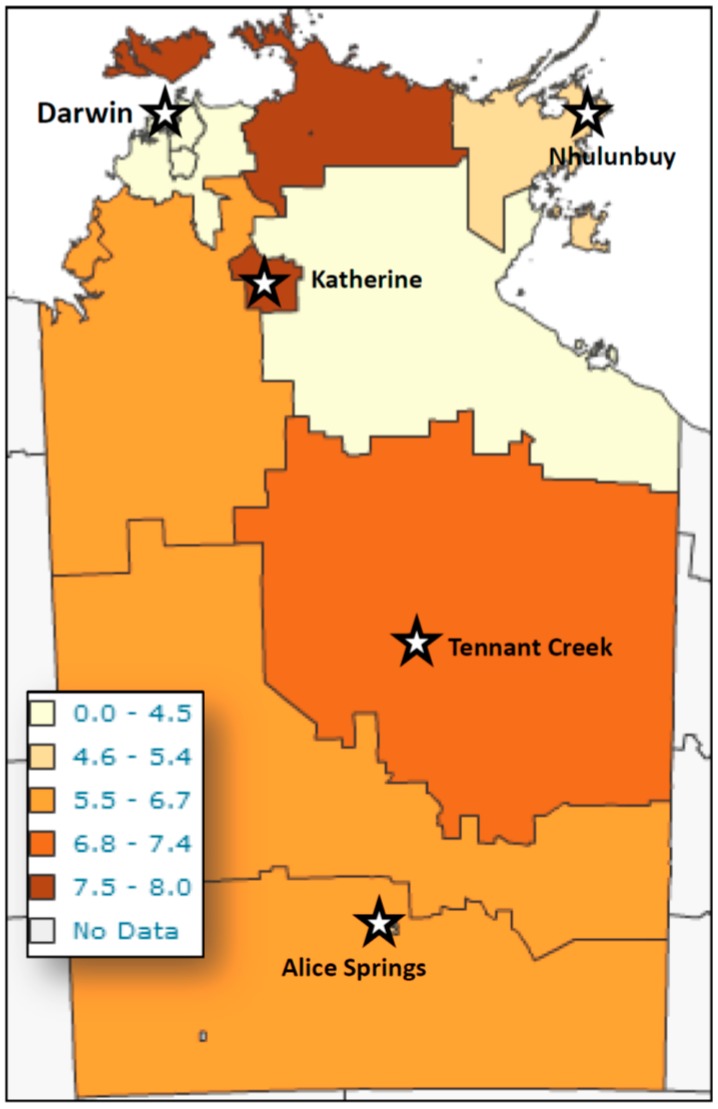
Geographic distribution of *S. stercoralis* larvae detection from patients in Northern Territory public healthcare facilities by geographic area of residence from 2002 to 2012. Prevalence figures are *S. stercoralis* larvae diagnostic detections in public health laboratories/10,000 Indigenous population/year.

Figure 1 is a cumulative graph of 11 years data presenting cases, and cases by number of faecal specimens examined. The age distribution of actual cases was biphasic (Figure 1), with the highest number of detections occurring in patients less than five years of age, with a peak in the second year of life, and a second smaller peak in the 35–50-year-old group. However, reviewing larvae detections to number of faecal specimens examined revealed the peak detection age in the 4–5 year-old age group. Cases were detected from across the NT, with the highest detection rates coming from West Arnhem, Tiwi Islands, and the Katherine regions (Figure 2).

The lowest detection rates were seen in the Darwin and the Gulf country, east of Katherine area.

**Figure 3:**
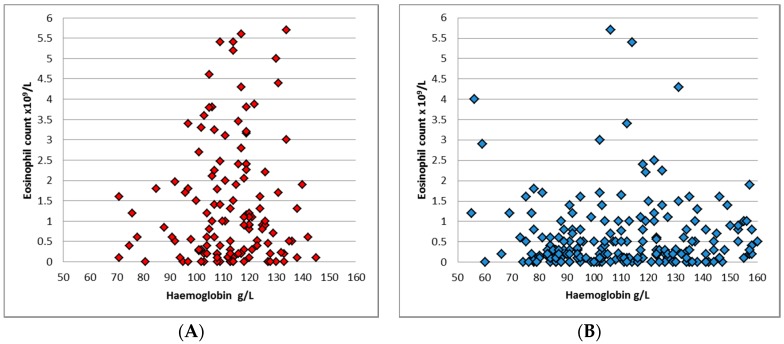
Distribution of contemporaneous haemoglobin and eosinophil counts in patients with *S. stercoralis* larvae detection. (**A**) Patients five-years-old and under (red diamonds represent the individual data points). (**B**) Patients aged older than five years of age (blue diamonds represent individual data points).

Anaemia was present in 164 (44.8%) of patients, with a median Hb of 113 g/L (interquartile range (IQR) 97.8–127) across both groups, and 114 g/L (IQR 105–122) and 111 g/L (IQR 91–134) in the younger and older groups respectively (*p* = 0.48). Anaemia was present in 38.6% of the younger group, compared to 49.1% in the greater than five-year-old cohort (*p* = 0.04). 184 (49.9%) patients had eosinophilia, with a median eosinophil count of 0.5 × 10^9^/L across both groups. Eosinophilia was more common in the younger group: 65.5% compared to 40.8% (*p* < 0.0001), and the median eosinophil count in ≤5 year-old cohort was 1 × 10^9^/L (Figure 3), while in >5 year-old group the median was 0.3 × 10^9^/L (*p* < 0.001).

A normal haemoglobin and normal eosinophil count was present in 19% of the younger group, and 29.6% of the older cohort. The combination of anaemia and eosinophilia was present in 22% of the ≤5 year-old age group, compared to 18% in the older cohort.

Polyparasitism was observed in 122 (31.4%) of all patients with *S. stercoralis* larvae detections. In the younger cohort 61 (37.2%) patients had at least one other parasite identified in their faecal specimen, while in the older group 61 (27.1%) of patients had at least one other parasite identified. Overall, 42 (10.8%) patients had co-infection with at least one other STH, 22 (5.6%) also had *Hymenolepis nana* identified, while 76 (33.8%) had co-infection with intestinal protozoa. 

**Figure 4:**
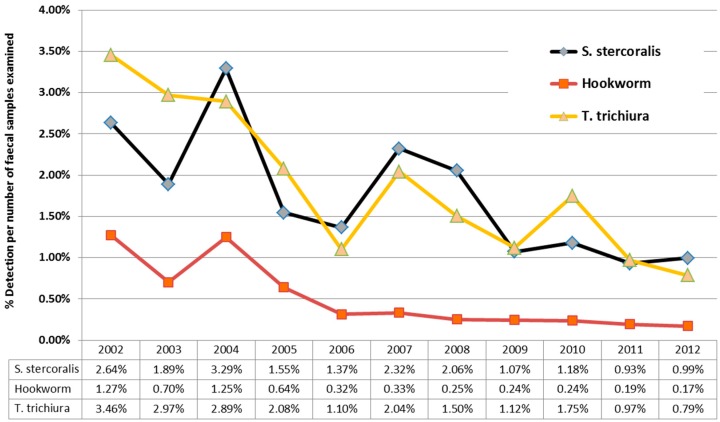
Comparative yearly detection of soil-transmitted helminths in the Northern Territory public laboratories, 2002 to 2012, by yearly diagnostic prevalence.

Over the 11 years of this study, there was a downward trend in *S. stercoralis* detections from 2.64% (in 2012) to 0.99% (in 2012) larvae detections/stool specimens examined. (Figure 4), mirroring the results for hookworm and *T. trichiura* over the same time period from the same cohort. This downward trend in detections was due to a three-fold reduction in detections in children under the age of five, while detection of larvae in patients over the age of five, remained relatively constant. 10,208 samples from children less than five were examined during this time period, with a median 882 samples/year (IQR 883–1003); with no significant variation in faecal numbers during the study period to account for the three-fold reduction.

## 3. Discussion

The main findings of this study include: (i) the declining rate of *S. stercoralis* detection over the 11 years of this study, which mirrors the previously noted decline of two other helminths, namely, hookworm, [11] and whipworm [12]; (ii) the biphasic age distribution of the patients with larvae detection; and (iii) *S. stercoralis* infections’ poor correlation with the presence of eosinophilia, particularly in detections in patients over the age of five, where eosinophilia was present in only 40.8% of patients.

Our findings are consistent with other studies of STH detection in the same time period [11,12] from the same cohort, revealing a declining detection rate in the NT. Two additional factors, possibly influencing the rates of detection of STHs in NT health care facilities have to be considered before definitely attributing the reduction to a community-wide decline in helminth infections. Firstly, hookworm has been targeted by an Indigenous community children’s deworming program (CCDP) in the NT since 1995, which administers single-dose albendazole to children aged 6 months to 16 years, twice a year [11]. This is a lower albendazole dose than current recommended for *S. stercoralis* treatment therapy, but may have a partial effect. The Central Australian Rural Practitioners Association (CARPA) [13] currently recommends for *S. stercoralis* therapy: albendazole daily for three days (children ≤5 years of age), or ivermectin, for children >5 years and adults. The albendazole given as part of the CCDP may still have some effect, as noted by decreases in the detection of whipworm [12] in the same population. Dwarf tapeworm (*Hymenolepis nana*), which is common, and not susceptible to albendazole, has not shown a temporal decrease in detection in the NT [14], from the same cohort, during this time period, suggesting the CCDP may possibly be associated with some additional STH reductions outside the targeted hookworm-infected population. Secondly, hospital paediatric admissions for diarrhea have reduced Australia-wide, since the introduction of rotavirus vaccine in 2006 [15]. This may have led to a decrease in detection of asymptomatic *S. stercoralis* infection in patients admitted with acute diarrhoeal diseases. However, the decreasing trend may reflect some improvement in socio-economic and sanitary circumstances, but this is speculative until formal prevalence studies are temporally conducted in a cross-section of the many remote communities spread across the 1.5 million square kilometers of the Northern Territory.

The biphasic age distribution of patients in raw detection numbers with a secondary age peak between 35 and 50 years of age, with a male predominance across the NT, is quite different to the pattern observed with whipworm [12] in the NT from the same cohort, where conversely, women had a statistically significant higher proportion of infections. It was postulated that adult women have higher rates of infection because they care for and live in closer proximity to infected children who contaminate the nearby environment. The adult male and female population is outside the target group for CCDP, so these infections detections do represent continuing NT wide endemic disease.

This study used a standard widely-used concentration method to detect *S. stercoralis* faecal larvae as the diagnostic, allowing the demographic and laboratory parameters in patients with current *S. stercoralis* infection to be accurately correlated with actual infection, rather than implied from serological results, that can remain positive after the infection is cleared [16]. The gold standard for the diagnosis of *S. stercoralis* remains serial stool examination [17]. However, traditional stool examinations are insensitive, and require up to seven stool exams to reach a sensitivity of 100%. Specialized stool exams include Harada-Mori filter paper culture, quantitative acetate concentration technique, and nutrient agar plate cultures [17]. Detection by nucleic acid amplification of faecal strongyloides DNA, is in development, but has not as yet been demonstrated to be superior to traditional methods for *S. stercoralis* infection [18,19] and is not yet widely available.

Of note in our study was the significant difference in eosinophilia observed between the ≤5 year-old group and the >5 year-old group. This observation supports local NT and Australia-wide guidelines endorsing ivermectin prophylaxis for all Indigenous adults undergoing immunosuppression, rather than just those with eosinophilia. Eosinophilia could also be more common in the younger age group related to factors such as more recent infection, presence of other nematodes or to a lesser extent, the cestode, *H. nana*. Our study emphasises that the presence or absence of eosinophilia is not an adequate proxy test for *S stercoralis* infection in a community where the infection is prevalent, particularly in patients over the age of five years. This finding supports an earlier study where *S. stercoralis* serology results and the presence of eosinophilia, were found to correlate poorly [20] though the correlation in patients with diabetes was somewhat better. The higher rates of anaemia, we noted in the Indigenous older age group has been documented previously, as anaemia is highly prevalent in Indigenous communities. A study in an Aboriginal community of Western Australia identified anaemia among 55% of women and 18% of men [21], the causes being multifactorial, and often associated with other comorbidities, including social disadvantage [22]. 

Our retrospective study has several limitations and several potential biases that require consideration when interpreting this data. This was not a formal prevalence study, as systematic sampling from individual communities was not undertaken, so the actual prevalence rates are undoubtedly much higher than the laboratory microscopically-diagnosed rates found in our study population. The study population mainly reflected inpatients of NT Government health facilities, reflecting a selection bias towards patients with acute illness and comorbid conditions. Bias may have been introduced in regards to Indigenous status (as Indigenous patients are over-represented amongst admissions to NT Health Care facilities) and the bias towards inclusion of hospitalised patients may have resulted in an inflated estimate of comorbid anaemia and co-infection. Reduced recovery of parasites due to delays incurred by transport of specimens to the laboratory from remote locations, and use of routine laboratory methods rather than specialised enrichment techniques, will have led to an underestimate of the actual number of infections. 

The main findings of this study of *S. stercoralis* larvae detections from NT Government health care facilities include: detections occur almost exclusively from Indigenous patients, across the entire NT; the detection of larvae in all age groups, the geographic distribution of larvae detection, and the lack of active infections being associated with eosinophilia. This last finding has important implications for patients receiving immunosuppressive therapy and their need for prophylactic *S. stercoralis* therapy. The temporal trend over the 11 years of this study, reveals declining rates of *S. stercoralis* detection, which mirrors that of the other helminths, whipworm, and hookworm, though all remain endemic in the NT, and remain markers of social disadvantage.

## 4. Materials and Methods

We conducted a retrospective observational review of microbiologically detected *S. stercoralis* larvae in faecal samples collected at NT Government Health facilities between 2002 and 2012 inclusive. Ethical approval for the study was obtained from both the Top End HREC-2013-1978 and Central Australian ethics committees, HREC-14-267. Cases were identified from the NT government pathology laboratory information system, Labtrak (Intersystems), which covers all NT Government Health facilities, including 5 hospitals, 2 correctional centres, and over 50 remote clinics. Previous STH studies have shown the NT public laboratories identified 94% of all documented STHs, as compared to 6% from other pathology providers, during this time period [11]. Given the intermittent shedding of *S. stercoralis* larvae, all faecal microscopy specimens (including second and third samples) were included in the analysis, but only one episode of larvae detection was recorded, even if multiple positive larvae detections were recorded in an individual episode of hospitalisation. Repeated episodes in a single patient were counted, if the temporal spacing was greater than six months. The episodes were linked to NT government electronic databases via medical record number to obtain data on age, sex, Indigenous status, geographic residence, haemoglobin level, and eosinophil count. Anaemia was defined as a haemoglobin <110 g/L and eosinophilia as a count ≥0.5 × 10^9^/L. Parasite identification was done by faecal specimen examination by wet mount microscopy followed by a formol ethyl-acetate concentration method [17]. Initially manual, the lab has also changed to use a proprietary product, namely Mini Parasep SF Faecal Concentrator (Apacor, Wolkingham, England). Local standard reporting procedures were followed. Quantitative assessment of parasite numbers was not performed. Detection of larvae allowed specific correlation of current infection with the haematological parameters, but is relatively insensitive compared to research methods [17].

## 5. Statistical Analysis

Data was collected in a Microsoft Excel 2010 (Microsoft Corporation, Redmond, Washington, DC, USA) database and analysed using the ‘Real-statistics’ plug in (Zaiontz C. 2015, www.real-statistics.com). Results are presented as medians and interquartile ranges (IQRs) for non-normally distributed parameters. Age range groups, and faecal detection rates were cumulated for presentation in Figure 1. Indigenous population data from the NT were obtained from Australian Bureau of Statistics data [23]. Bivariate analyses were performed using the Chi Square or Fisher exact test (if expected frequencies were less than 5). For non-parametric data, (median haemoglobin and eosinophil counts, as well as polyparasitism in patients above and below the age of 5 years), the Mann–Whitney *U* test was used, with *p* values of <0.05 considered significant. The age cut-off of five years was chosen based on previous studies of STH in a similar population [11].

## Figures and Tables

**Table 1:** Demographic and laboratory parameters of patients with *S. stercoralis* larvae detection in the Northern Territory, 2002 to 2012.

Parameter	≤ 5 Years Old (%)	> 5 Years Old (%)	All Ages (%)	*p*-Value
**Number ^1^**	164 (42.2)	224 (57.6)	389 (100)	
**Sex**				
Male	79 (48.2)	137 (60.9)	216 (55.5)	0.012
Female	85 (51.8)	88 (39.1)	173 (44.5)	
**Indigenous status**				
Indigenous	163 (99.4)	218 (96.9)	381 (97.7)	<0.0001
Non-indigenous	1 (0.6)	7 (3.1)	8 (2.3)	
**Laboratory parameters ^2^**				
Median haemoglobin	114 (IQR 105–122)	111 (IQR 91–134)	113 (IQR 97.8–127)	0.48
Anaemia ^3^	58 (38.6)	106 (49.1)	164 (44.8)	0.04
Median eosinophil count	1 (IQR 0.2–2.4)	0.3 (IQR 0.1–0.8)	0.5 (IQR 0.1–1.3)	<0.0001
Eosinophilia ^4^	95 (65.5)	89 (40.8)	184 (49.9)	<0.0001
**Polyparasitism ^5^**	61 (37.2)	61 (27.1)	122 (31.4)	0.03
Other intestinal helminths	13 (7.9)	29 (12.9)	42 (10.8)	0.12
*Hymenolepis nana*	18 (11)	4 (1.8)	22 (5.6)	<0.001
Other (eg. Protozoa)	40 (24.4)	36 (16)	76 (33.8)	0.04

^1^ One Indigenous male, did not have age or haematological data. ^2^ 23 patients (4.3%) had no haematological data, of these 14 were ≤ 5 years old. An additional 6 patients had no data for eosinophilia (5.4%), 5 were ≤ 5 years old. ^3^ Anaemia: Haemoglobin ≤110 g/L. ^4^ Eosinophilia: eosinophils > 0.5 × 10^9^ /L. ^5^ Intestinal helminths were hookworm and *T. trichiura*. *Ascaris* species were not detected.
